# Supplementary material for: The impact of land reform on the status of large carnivores in Zimbabwe
Source: PeerJ. 2016 Jan 14;4:e1537. doi: 10.7717/peerj.1537 (PMC4728035; doi:10.7717/peerj.1537)
Supplement: Supplemental Information 1 [file peerj-04-1537-s001.pdf]

Supplementary Information - Article S1

The Impact of Land Reform on the Status of Large Carnivores: a preliminary assessment of national population trends of large carnivores in Zimbabwe

Samual T. Williams, Kathryn S. Williams Christoffel J. Joubert, Russell A. Hill

Corresponding author: Samual T. Williams, s.t.williams@durham.ac.uk.

Introduction

The importance of private land to wildlife conservation is becoming increasingly clear (Bond *et al*., 2004). Species such as cheetahs (*Acinonyx jubatus*) were thought to depend heavily on private land as they are out competed in protected areas by larger carnivores (Durant, 1998), for example 80% of cheetahs in Zimbabwe occurred on private land (Stuart & Wilson, 1988). Since 2000, however, most private land in Zimbabwe has been rapidly resettled under the fast-track land reform programme (FTLRP), resulting in large scale settlement of private land (Scoones *et al*., 2010), a process which has huge potential to impact the population of large carnivores. This supplementary information considers the impact of fast-track land reform on the population trends of large carnivores in Zimbabwe through extrapolating our findings from Savé Valley Conservancy (SVC) (Williams *et al*. (in review) to a national scale, based on the assumption that the trends observed following resettlement at SVC are representative across the country.

Large carnivores were recorded at much greater densities in the private land use type (LUT) than the resettlement or communal LUTs in south east Zimbabwe (Williams *et al*., in review). Cheetah, lion (*Panthera leo*), leopard (*Panthera pardus*), brown hyaena (*Parahyaena brunnea*) and African wild dog (*Lycaon pictus*) were present only in the private LUT, where they occurred at similar densities to protected areas (Williams *et al*., in review). Spotted hyaena (*Crocuta crocuta*) was the only species to occur in the resettlement LUT, but their density was 7.4 times greater in the private LUT than the resettlement LUT (Williams *et al*., in review). No large carnivore sign were recorded in the communal LUT. The study site constituted approximately 10.3% of the remaining private land in Zimbabwe, so it is reasonable to extrapolate these findings to a national scale.

Methods

The impact of the FTLRP on carnivore population sizes on private land at a national level across Zimbabwe was estimated using the following linear model:

$$P_{2008} = (P_{previous} \text{ x } A_{resettled} \text{ x } C_{remaining}) + (P_{previous} \text{ x } A_{remaining})$$

Where P_{2008} and $P_{previous}$ represent the 2008 and previous (prior to resettlement in 2000) population sizes of each study species on private land in Zimbabwe respectively.

$A_{resettled}$ represents the proportion of private land that has been resettled between 2000 and 2008, while $A_{remaining}$ represents the proportion of private land remaining in 2008. $C_{remaining}$ represents ratio of the density carnivores that that occur on resettlement land to the density of carnivores that occur on private land.

Estimates of carnivore population size on private land and in total in Zimbabwe before 2000 were taken from the literature where available. Populations on private land were assumed to have remained stable within each LUT between 2000 and 2008. Estimates of brown hyaena abundance in the literature were not broken down by land use type, and no estimates were available for the proportion of the population that was thought to occur on private land. For this species the number of individuals on private land was estimated by multiplying the total estimate by the proportion of the species range in Zimbabwe (excluding communal land) that was composed of private land, which was calculated by digitizing a map of land use type (Surveyor-General, 1998) using QGIS 2.8.2 (QGIS Development Team, 2015).

Results

The estimated total population size of large carnivores in Zimbabwe after the FTLRP differed significantly from population size before the FTLRP (Wilcoxon matched pairs: $Z = 0.000$, $df = 5$, $P = 0.028$; Table 1). The density of each study species was lower after the FTLRP than before. The most dramatic decline was calculated for cheetahs, which were estimated to have declined by approximately 70%. Steep declines were also estimated for leopards (58%-69%) and brown hyaenas (47%) as a result of the FTLRP. Wild dogs and spotted hyaenas displayed more modest declines (29% and 11% respectively), while lion are estimated to have declined by only 2%. Across all species the mean change in population size was a 37% decline (when using average cheetah and leopard maximum and minimum estimates).

Discussion

When extrapolated to a national scale, these data suggest that changes in land use associated with the FTLRP resulted in a decline in the population size of each large carnivore species between 2000 and 2008. The largest impact of resettlement was on species that used to have large proportions of their populations occurring on private land, such as cheetahs. The estimated 70% decline of the cheetah population size in Zimbabwe over eight years is a much steeper decline than the suspected 30% decline in the global cheetah population between 1992 and 2010 (Durant *et al.*, 2008). Leopards, brown hyaenas and wild dogs also had substantial populations on private land, and their numbers were estimated to have declined by 29-69%. The relatively moderate 11% decline for spotted hyaena and 2% for lion can be explained by the fact that private land supported a much smaller proportion of their national population.

It is suggested that further research is conducted at other sites to determine if these findings are representative of population trends of large carnivores across Zimbabwe. Further studies in other countries undergoing land reform programmes would also help to determine if these trends are representative internationally.

References

Bauer H, and Van Der Merwe S. 2004. Inventory of free-ranging lions *Panthera leo* in Africa. *Oryx* 38:26-31.

Bond I, Child B, de la Harpe D, Jones B, Barnes J, and Anderson H. 2004. Private land contribution to conservation in South Africa. In: Child B, ed. *Parks in transition: biodiveristy, rural development, and the bottom line*. London: Earthscan, 29-61.

Chardonnet P. 2002. Conservation of the African lion: contribution to a status survey, International Foundation for the Conservation of Wildlife, France and Conservation Force, USA.

Davidson Z, and Loveridge A. 2006. The Gwai conservancy: lion density and quota recommendations based on spoor density estimation. Wildlife Conservation Research Unit, University of Oxford, Oxford.

Davison B. 1999. An estimate of the status of the cheetah in Zimbabwe. Zimbabwe Department of National Parks and Wildlife Management, Harare.

du Toit R. 2004. Review of wildlife issues associated with the land reform programme in Zimbabwe. World Wide Fund for Nature (WWF), Southern African Regional Programme Office, Harare.

Durant S, Marker L, Purchase N, Belbachir F, Hunter L, Packer C, Breitenmoser-Wursten C, Sogbohossou E, and Bauer H. 2008. *Acinonyx jubatus*. IUCN red list of threatened species. Version 2015.2. *Available at* http://www.iucnredlist.org/apps/redlist/details/219/0 (accessed 14 July 2015).

Durant SM. 1998. Competition refuges and coexistence: an example from Serengeti carnivores. *Journal of Animal Ecology* 67:370-386.

Jacquier M, and Woodfine T. 2007. A pilot GPS study on a single male cheetah in Zimbabwe. *Cat News* 47:15-17.

Martin RB, and de Meulenaer T. 1988. Survey of the status of the leopard (*Panthera pardus*) in sub-Saharan Africa. Secretariat of the Convention on International Trade in Endangered Species of Wild Fauna and Flora, Lausanne.

Mills G, and Hofer H. 1998. Hyaenas: status survey and conservation action plan. IUCN/SSC Hyaena Specialist Group, Gland.

Myers N. 1975. *The cheetah, Acinonyx jubatus, in Africa*. *IUCN Monograph 4*. Morges: International Union for Conservation of Nature and Natural Resources.

Pole AJ. 2000. The behaviour and ecology of African wild dogs, *Lycaon pictus*, in an environment with reduced competitor density. PhD thesis. University of Aberdeen.

QGIS Development Team. 2015. Quantum GIS Geographic Information System [computer program]. Available from http://qgis.org/en/site/. Open Source Geospatial Foundation Project.

Scoones I, Marongwe N, Mavedzenge B, Mahenehene J, Murimbarimba F, and Sekume C. 2010. *Zimbabwe's land reform: myths & realities*. Woodbridge: James Currey.

Stuart CT, and Wilson VJ. 1988. *The cats of southern Africa*. Bulawayo: The Chipangali Wildlife Trust.

Surveyor-General. 1998. Zimbabwe land classification Surveyor-General, Harare.

White J. 1996. Survey of cheetah and leopard population on commercial farms. The Wildlife Producers Association, Harare.

Williams ST, Williams KS, Joubert CJ, and Hill RA. in review. The Impact of land reform on the status of large carnivores in Zimbabwe. *PeerJ*.

Wilson VJ. 1984. The distribution and present day status of the felids of Zimbabwe. Chipangali Wildlife Trust, Bulawayo.

Wilson VJ. 1997. Biodiversity of Hwange National Park, Zimbabwe. Chipangali Wildlife Trust, Bulawayo.

Woodroffe R, McNutt JW, and Mills MGL. 2004. African wild dog *Lycaon pictus* (Temminck, 1820). In: Sillero-Zubiri C, Hoffmann M, and Macdonald DW, eds. *Canids: foxes, wolves, jackals and dogs Status survey and conservation action plan*. Gland: IUCN/Species Survival Commission Canid Specialist Group.

Tables

Table 1. Population size estimates for large carnivores in Zimbabwe in 2000 and 2008.

		Private land		Other land[a]	Total		
Species	Proportion of private population remaining on resettled land	Population size in 2000	Population size in 2008	Population size in 2008[b]	Population size in 2000	Population size in 2008	Change in population size between 2000 and 2008 (%)
Cheetah (minimum)	0.00	320[c]	42	80[c]	400[d]	122	-70
Cheetah (maximum)	0.00	1,200[e]	156	320[e]	1,520[e]	476	-69
Leopard (minimum)	0.00	1,579[f]	205	421[g]	2,000[h]	626	-69
Leopard (maximum)	0.00	10,745[i]	1,397	5,319	16,064[i]	6,716	-58
Lion	0.00	31[j]	4	1,597[j]	1,628[j]	1,601	-2
Wild dog	0.00	200[k]	26	400[l]	600[l]	426	-29
Brown hyaena	0.00	54[m]	7	46[m]	100[m]	53	-47
Spotted hyaena	0.14	800[n]	198	4,800[n]	5,600[n]	4,998	-11

[a]Other land is defined as any land use type other than private. This includes state protected areas such as national parks, safari areas and forestry land; and communal land. [b]Assuming that carnivore populations outside private land have remained stable between 2000 and 2008; [c]Of 400 cheetahs in Zimbabwe (Myers, 1975) 80% occurred on private land (Stuart & Wilson, 1988); [d](Myers, 1975); [e](Davison, 1999); [f](White, 1996); [g]Calculated by subtracting 1,579 animals on private land (White, 1996) from a total of 2,000 animals (Wilson, 1984); [h]Wilson (1984); [i](Martin & de Meulenaer, 1988), assuming that all unmodified land is made up of state-protected areas and private land and that all modified land is communal land; [j]See Table 2 for details of how this was calculated; [k](Woodroffe, McNutt & Mills, 2004), assuming that all wild dogs occurring outside protected areas were on private land; [l](Woodroffe, McNutt & Mills, 2004); [m](Mills & Hofer, 1998), assuming that the brown hyaenas in Zimbabwe are absent from communal land (Table 3) but otherwise occur at an equal density throughout their range. After excluding communal land, private land makes up 54% of remaining brown hyaena range in Zimbabwe; [n](Mills & Hofer, 1998).

Table 2. Sources and calculations used to generate estimates of lion population sizes.

	Estimated number of lions in Zimbabwe before 2000					
	From literature		Used in analysis			
Region (following (Chardonnet, 2002))	Estimate from (Chardonnet, 2002) (includes all land use types)	Estimate from (Bauer & Van Der Merwe, 2004) (generally includes protected areas only)	Private	State protected areas (PAs)	Communal (CL)	Sources used, and how estimate used in analysis calculated
Mana Pools NP & surrounding SAs and CLs	495	442	N/A	442	53	For PAs used (Bauer & Van Der Merwe, 2004). For communal used estimates of (Bauer & Van Der Merwe, 2004) were subtracted from estimates of (Chardonnet, 2002).
Matusadona & Chizarira NPs, surrounding SAs & CL	310	260	N/A	260	50	For PAs used (Bauer & Van Der Merwe, 2004). For CL used total estimate (310 lions; (Chardonnet, 2002)) minus PA estimate (260 lions; (Bauer & Van Der Merwe, 2004)).
Hwange NP	543	120	N/A	543	N/A	Used (Chardonnet, 2002). Estimate of 120 lions (Bauer & Van Der Merwe, 2004) seems low. 220 known individuals in Hwange NP up to 2004 (Davidson & Loveridge, 2006). At least 1,000 lions listed in (Wilson, 1997).
Matetsi complex (including NPs, SAs & FL) & Gwayi complex	150	85	20	130	N/A	Private estimate of 20 lions in Gwayi from (Davidson & Loveridge, 2006) (assuming lion population has remained stable 2000-2006). For PA estimate (Bauer & Van Der Merwe, 2004) only provide estimates for Matetsi SA (60) and Zambezi NP (25), so instead used estimate (150 lions in total) from (Chardonnet, 2002) as this source provided more comprehensive coverage. Total (150 lions; (Chardonnet, 2002)) minus private (20 lions) leaves 130 lions in protected areas.
Gonarezhou NP, Malipati SA & conservancies	183	130	11	114	N/A	Private estimate (11 lions) assumes lion density from (Pole, 2000) (0.24/100km^2) for Savé Valley Conservancy (3,440 km^2) also applies to Chiredzi River Conservancy (800km^2 (du Toit, 2004)) and Malilangwe (400 km^2, (Jacquier & Woodfine, 2007)). Estimate of (130 lions; (Bauer & Van Der Merwe, 2004)) included Gonarezhou NP, Savé Valley Conservancy, Chiredzi River Conservancy, Malilangwe, Beitbridge and Tuli SA. PA estimate calculated by subtracting private estimate (11 lions) and Tuli SA estimate (5 lions) (Chardonnet, 2002) from total estimate of 130 lions (Bauer & Van Der Merwe, 2004), leaving 114 lions.
Tuli SA	5	Included in Gonarezhou NP estimate	N/A	5	N/A	Used (Chardonnet, 2002).
**Total**	**1686**	**1037**	**31**	**1494**	**103**	

Abbreviations:
NP - National Park
SA - Safari Area
FL - Forestry land
PA - state protected areas (NPs, SAs & FL)
CL - Communal land
